# THE RELATIONSHIP BETWEEN SPIRITUALITY AND COGNITION WITHIN BLACK AND WHITE ADULT IN THE HANDLSLEEP STUDY

**DOI:** 10.1093/geroni/igae098.0633

**Published:** 2024-12-31

**Authors:** Marsha Hampton, Alexa Allan, Linying Ji, Lesley Ross, Roland Thorpe, Alan Zonderman, Michele Evans, Alyssa Gamaldo

**Affiliations:** Clemson University, Clemson, South Carolina, United States; The Pennsylvania State University, University Park, Pennsylvania, United States; Montana State University, Bozeman, Montana, United States; Clemson University, Clemson, South Carolina, United States; Johns Hopkins University, Baltimore, Maryland, United States; National Institute on Aging, Baltimore, Maryland, United States; National Institute on Aging, Baltimore, Maryland, United States; Clemson University, Clemson, South Carolina, United States

## Abstract

This study explored associations between spirituality and cognitive performance within Black and White adults in the HANDLS Sleep Study (n=85; 52% Black adults; age range: 46-82). The Spirituality Transcendence Scale-Short Form measured overall spiritual transcendence orientation and three subscale scores (Prayer Fulfillment, Universality, and Connectedness). Cognitive performance was measured by several tests (MoCA, Trails Making Test, AVLT, Digit Span, Clock Drawing, Verbal Fluency). Race-stratified regression analyses explored the association between each spirituality score and cognitive test after covariate adjustment (age, sex, poverty status, depression severity and health conditions). Within Black adults, the spiritual subscale (universality and connectedness) and total scores were significantly associated with better delayed AVLT (memory; b = -.409, p = 0.014), phonemic verbal fluency (executive function; b = 1.312, p =.044) and clock drawing (visuospatial skill; b = 0.092, p = 0.040). However, these findings became non-significant after covariate adjustment. Within White adults, results indicated that higher spiritual personal fulfillment (b = -.281, p = 0.014) was significantly associated with worse performance on digit span forward (executive function); however, higher connectedness (b =.442, p = 0.014) was significantly associated with better performance on the same test after covariate adjustment. Higher connectedness was significantly associated with faster performance on Trials B (b = -4.452, p = 0.015; executive function), but was non-significant after covariate adjustment. Findings illustrate differential associations between the spirituality and cognitive performance within Black and White adults, which suggest further exploring psychosocial factors culturally relevant to each racial group to understand current findings.

